# An evaluation of gemcitabines differential radiosensitising effect in related bladder cancer cell lines

**DOI:** 10.1038/sj.bjc.6601538

**Published:** 2004-01-20

**Authors:** V K Sangar, R Cowan, G P Margison, J H Hendry, N W Clarke

**Affiliations:** 1 Cancer Research UK Experimental Radiation Oncology Group, Paterson Institute for Cancer Research, Manchester M20 4BX, UK; 2 Christie Hospital NHS Trust, Manchester M20 4BX, UK; 3 Cancer Research UK Carcinogenesis Group, Paterson Institute for Cancer Research, Manchester M20 4BX, UK; 4 Salford Royal Hospitals NHS Trust, Salford M6 8HD, UK

**Keywords:** gemcitabine, bladder, radiation

## Abstract

The aim of this study was to establish the radiosensitising properties of gemcitabine in a pair of related bladder tumour cell lines with differential radiosensitivity. The radioresistant bladder tumour cell line MGH-U1 and its radiosensitive mutant clone, S40b (both p53 mutant), had SF_2_ values (surviving fraction at 2 Gy) of 0.98 and 0.64, respectively (*P*<0.001). Colony-forming assays showed that at 0.01 *μ*M gemcitabine radiosensitisation occurred only in the S40b cell line (dose-modifying factor (DMF)=1.4). At 0.3 *μ*M (killing 50% of cells), both cell lines were radiosensitised; DMF=2.25 and 1.2 for MGH-U1 and S40b, respectively. These data suggest that gemcitabine is an effective radiosensitiser in bladder cancer cell lines, with greater sensitisation in the radioresistant parental line–a feature that should be useful in a clinical setting.

The incidence of bladder cancer is estimated at 13 000 cases per annum in the UK (Office of Population Censuses and Surveys, 1995). Of these, approximately 20% are invasive transitional cell carcinomas (TCC) (T2+). Radical radiotherapy as a treatment for invasive TCC of the bladder is well established. The overall 5-year survival rates are 24–29% (Blandy *et al*, 1980; Fossa *et al*, 1993), a figure lower than in some surgical series (Skinner *et al*, 1991). However, in some reported series, radiotherapy has overall 5-year survival figures similar to radical cystectomy (Jenkins *et al*, 1988). Although both treatments have comparable morbidity and mortality, there is one fundamental difference: with radiotherapy, the patient retains a functioning natural bladder and males will usually retain erectile function. Thus, radiotherapy has greater potential to help retain better quality of life after treatment.

The lower survival following radiotherapy in some series *vs* others can be attributed to the differing degrees of heterogeneity among the tumours or to the inadequate delivery of radiation. Chemotherapeutic agents that have the ability to radiosensitise tumours may result in more promising outcomes for TCC treated with radiotherapy.

The pyrimidine analogue gemcitabine has been studied as a radiosensitiser in a variety of preclinical models including colon, pancreas, head and neck, lung and mammary tumour cells (Shewach *et al*, 1994; Lawrence *et al*, 1996; Mason *et al*, 1999; Pacini *et al*, 1999; Fields *et al*, 2000; Robinson and Shewach, 2001). These studies showed that chemoradiation using gemcitabine produced radiation dose-modifying factors (DMF) of between 1.1 and 3 using various combinations of drug and radiation. The radiosensitising effects were dependent on dose, time of administration and tumour cell type. Effective radiosensitising doses could be as little as 0.1 *μ*M (a noncytotoxic dose), even using short incubation times (4 h), but the radiosensitising effects tended to plateau at higher cytotoxic drug levels. Furthermore, the highest value of DMF was seen when gemcitabine was administered at least 24 h prior to irradiation.

There have been studies using TCC cell lines that have shown conflicting results. Fechner *et al* (2003) studied four different TCC cell lines (RT112, RT4, T24 and SUP) with differing p53 status and found that the addition of gemcitabine resulted in no radiosensitising effect. Another study (Pauwels *et al*, 2003) investigated a number of tumour cell types including an epidermioid bladder cancer cell line (ECV304) and found significant radiosensitising effects caused by gemcitabine. They found dose enhancement factors of 1.39–3.05 depending on the concentration used. It is likely that these differences are due to the experimental technique. Neither study utilised the standard colony-forming assay, which is the accepted principal technique providing data with direct implications for radiotherapy efficacy regarding tumour control. Further work with TCC cell lines is therefore essential if this agent is to be used clinically.

In an attempt to understand how gemcitabine radiosensitises, its effects have been studied in relation to a number of factors including dATP reduction, alteration of DNA repair, cell cycle perturbations, deoxycytidine kinase levels and apoptosis (Shewach *et al*, 1994; Joschko *et al*, 1997; Gregoire *et al*, 1998, 2002; Milas *et al*, 1999; Ostruszka and Shewach, 2000; Lawrence *et al*, 2001; Van Putten *et al*, 2001). Chen *et al* (2000) postulated that the radiosensitising effects of gemcitabine might be related to p53 status. However, they concluded in their study on RKO-E6 and RKO-P colon cancer cell lines, which differed in p53 status, that it was not the single most important factor. Later, Robinson and Shewach (2001) studied related MCF-7 breast cancer cell lines with different p53 status and found no difference in radioenhancement by dFdC.

The study of related cell lines and the differential effects of treatments upon them may increase our understanding regarding the molecular mechanisms that increase or decrease survival. There are few such TCC models available. The MGH-U1 cell line can be regarded as a standard TCC cell line that is known to be relatively radioresistant (McMillan and Holmes, 1991). Utilising mutagenic and limiting dilution techniques, the S40b cell line was isolated from MGH-U1. This mutant clone has previously been shown to be significantly more radiosensitive than the parent MGH-U1 cell line and together they provide a valuable study model. Therefore, the aims of our study were to investigate the radiosensitising effects of gemcitabine in these two related TCC cell lines in relation to both p53 and cell cycle perturbations.

## METHODS

### Cell lines

The TCC cell lines (MGH-U1 and its subclone S40b) and a human fibroblast cell line (HF19) were grown as monolayers in HAMS F12 culture medium (containing 1 mM L-glutamine, GibcoBRL) supplemented with 10% foetal calf serum, 100 IU ml^−1^ penicillin and 0.1 mg ml^−1^ streptomycin (GibcoBRL). All cultures were kept at 37°C in a mixture of 3% O_2_, 5% CO_2_ and 92% N_2_. They were constantly checked for *Mycoplasma* spp infection, which remained absent. Cells were subcultured regularly to ensure exponential growth.

### Gemcitabine dose–response

The micro-tetrazolium assay (MTT) [3-(4,5-dimethylthiazol-2yl)-2,5-diphenyl-tetrazoliumbromide], optimised for MGH-U1 and S40b, was used to assess the gemciatbine dose–response curve. Exponentially growing cells (200 MGH-U1 cells and 400 S40b cells) in 100 *μ*l media were plated in 96-well plates (Helena BioSciences, Sunderland, UK). Cells were incubated for 24 h to allow adherence after which they were treated for 4 h with varying doses of gemcitabine (0–10 *μ*M) (Gemzar, Eli Lilly & Co., Basingstoke, UK). A stock solution of gemcitabine (1000 *μ*M in PBS) was diluted to appropriate concentrations in Hams F12 media containing 1 mM L-glutamine (GibcoBRL, Paisley, UK) and supplemented with 10% foetal calf serum. After exposure, cells were washed with phosphate-buffered saline (PBS) and fresh medium was added. Following incubation for a further 7 days, 50 *μ*l of 1 mg ml^−1^ 3-(4,5-dimethylthiazol-2yl)-2,5-diphenyl-tetrazoliumbromide in PBS was added to each well. Cells were incubated for 5 h at 37°C, then the medium was aspirated and the resultant formazan crystals were resuspended in 200 *μ*l of DMSO. Plates were read at 540 and 690 nm on a Multiskan platereader (Flow Ltd, Ayrshire, UK). Growth inhibition was calculated using an Excel MTT program (written by Dr Tim Ward, Drug Development and Imaging Group, Paterson Institute for Cancer Research, UK), which calculates the growth of treated cells as a fraction of that for the untreated control cells. The data were then plotted and analysed using unweighted nonlinear least-squares regression to identify significant differences between the two dose–response curves. The doses of gemcitabine resulting in 10–20% (IC_10–20_) and 50% (IC_50_) cell kill were derived from the curves.

### Radiation alone and in combination with gemcitabine

To assess cell kill after radiation alone and in combination with gemcitabine, subconfluent cell monolayers of MGH-U1 and S40b were trypsinised and diluted to form a single cell suspension of 1 × 10^5^ cells ml^−1^, which was then gamma-irradiated on ice (1 Gy min^−1^). After irradiation, the cells were diluted, so that the appropriate number of cells were seeded into 60 mm culture dishes in order to give at least 50 colonies. At 10 days after irradiation, colonies comprising at least 50 cells were stained with gentian violet and then counted.

For chemoradiation experiments, cells were incubated for 4 h with the IC_10–20_ and IC_50_ concentrations of gemcitabine 12 h prior to irradiation. Experiments were repeated in order to provide at least three sets of independent data, which were fitted using the linear quadratic model.

The number of colonies surviving 2 Gy of irradiation (SF_2_) was taken as a measure of radiosensitivity. Enhancement of the radiation response by gemcitabine (synergy) was defined as any effect on cell kill that was greater than would be expected from addition of the effects of the two agents alone. Analysis of variance and the F-test were used to compare the dose–response curves, which were fitted to the linear quadratic model, for irradiation alone and irradiation and gemcitabine combined.

### Cell cycle changes with single and combination treatment

MGH-U1 and S40b cells were plated into tissue culture flasks and given 2 Gy irradiation, IC_50_ gemcitabine, both IC_50_ Gemcitabine and 2 Gy, or no treatment. At various time points after treatment (0–48 h) flasks were trypsinised and cells were collected by centrifugation, resuspended in 200 *μ*l PBS and fixed in 2 ml ice cold 70% ethanol at 4°C for at least 30 min. The cells were collected by centrifugation and resuspended in a mixture of PBS (400 *μ*l), 1 mg ml^−1^ RNase (100 *μ*l) and 400 *μ*g ml^−1^ of propidium iodide (50 *μ*g) before incubating at 37°C for 30 min. Cell cycle analysis was carried out using a Becton Dickinson FACScan flow cytometer at 488 nm. For each treatment, the changes were calculated in the percentage of cells in each part of the cell cycle (G0/1, S and G2/M) compared to untreated controls.

### p53 functional analysis

The cell lines MGH-U1 and S40b along with HF19 (positive control) were analysed for the presence of p53. Approximately 2 × 10^5^ cells were plated and grown to ensure exponential growth. Cultures were then washed twice with PBS (5 ml) and then scraped in PBS (5 ml) and centrifuged (390 g) for 5 min. Pellets were resuspended in 1 ml of PBS and transferred to Eppendorf tubes before centrifuging (11 600 g) for 2 min. The cells were resuspended in 100 *μ*l of paraformaldehyde (4%) and left at room temperature for 30 min then washed twice in PBS by centrifugation before resuspending in 100 *μ*l of permeabilisation buffer (Saponin) containing p53 mouse monoclonal antibody (1 : 50) (D0-7, Novocastra, UK) and incubated for 60 min at 37°C. The cells were then washed twice in permeabilisation buffer before adding 100 *μ*l of buffer containing fluorescent-tagged rabbit antimouse antibody (1 : 40) (DAKO Ely, UK) and left to stand at room temperature for 45 min. After washing twice in PBS, by centrifugation cells were resuspended in a mixture of PBS (400 *μ*l), 1 mg ml^−1^ RNase (100 *μ*l) and 400 *μ*g ml^−1^ of propidium iodide (50 *μ*g) before incubating for 30 min at 37°C. As a negative control the above process was repeated, but omitting the addition of the p53 antibody. Cell suspensions were analysed by flow cytometry (Becton Dickinson FACScan) at 488 nm. The relative fluorescence was calculated by normalising against negative controls. Experiments were carried out on two independent occassions. The Student's *t*-test was used to detect any significant difference between the two cell lines.

The functional status of p53 was established by the assessment of cell cycle arrest after 2 Gy (see above).

## RESULTS

### Gemcitabine dose–response

**Figure 1:**
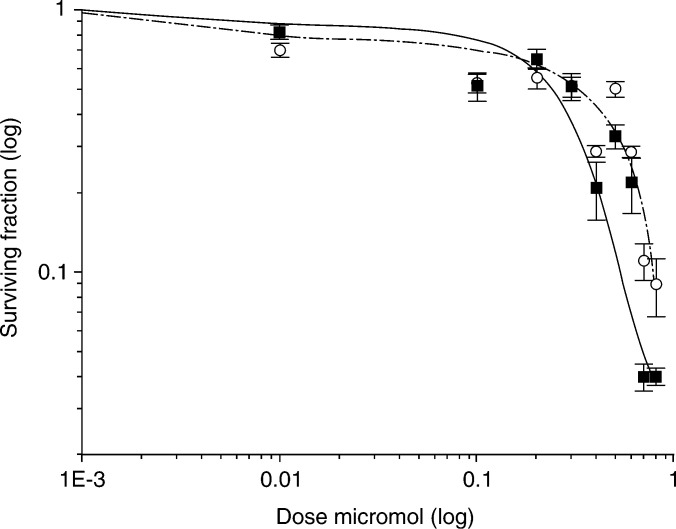
Dose–response curves for MGH-U1 (squares) and S40b (circles and dashed line) cell lines treated with dFdC. Data were obtained from three repeat MTT experiments.

The dose–response curves for MGH-U1 and S40b when treated with gemcitabine are shown in Figure 1

. There was no statistical significance between the response of the two cell lines. The IC_10–20_ was approximately 0.01 *μ*M for each cell line, while the IC_50_ was 0.25 and 0.35 *μ*M for S40b and MGH-U1, respectively. The doses used for further experiments were 0.01 *μ*M (IC_10-20_) and 0.3 *μ*M (average IC_50_).

### Radiation dose–response

**Figure 2:**
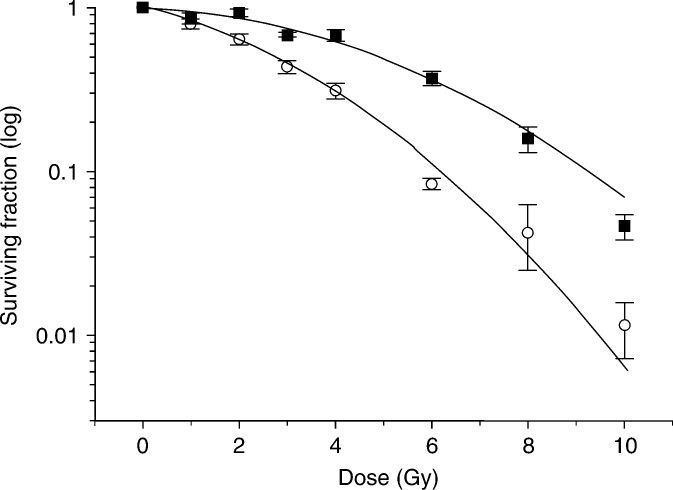
Clonogenic radiation survival curves for MGH-U1 (squares) and S40b (circles). Data from three independent experiments were averaged and fitted to the linear quadratic model. SF_2_ for MGH-U1=0.98±0.09 and S40b=0.64±0.01.

Using the colony–forming assay and fitting the data to the linear quadratic model, a significant difference was observed between the radiosensitivities of both cell lines. The surviving fraction at 2 Gy (SF_2_) was 0.98±0.09 and 0.64±0.01 for MGH-U1 and S40b, respectively (*P*<0.001) (Figure 2

).

### Radiation with gemcitabine

**Figure 3:**
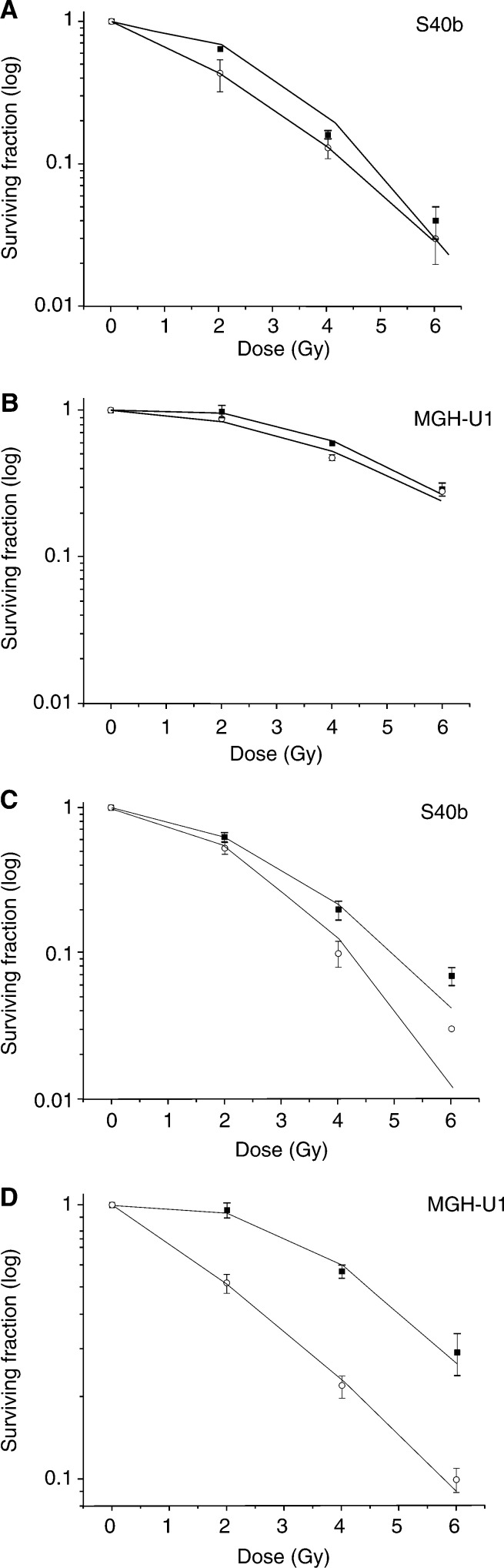
**(A–D)** Clonogenic survival curves for cells given irradiation alone (squares) and gemcitabine (4-h incubation) 12 h prior to irradiation (circles); 0.01 *μ*M gemcitabine in S40b (**A**) and MGH-U1 (**B**); 0.3 *μ*M gemcitabine in S40b (**C**) and MGH-U1 (**D**). Dose modification factors (DMF) were calculated at IC_50_ and were 1.4 (*P*<0.01), 1.1 (not significant), 1.2 (*P*<0.01) and 2.25 (*P*<0.001) for **A**, **B**, **C** and **D**, respectively.

When gemcitabine was given for a 4-h incubation commencing 12 h prior to irradiation, 0.01 *μ*M (IC_10–20_) radiosensitisation occurred in the radiosensitive S40b cell line (DMF=1.4; *P*<0.01), while in the MGH-U1 cell line, there was no significant change from irradiation alone (DMF=1.1) (Figure 3A, B

). At 0.3 *μ*M (IC_50_) both cell lines were radiosensitised, but the effect was considerably greater in the radioresistant MGH-U1 cell line (DMF=2.25; *P*<0.001) than in the radiosensitive S40b cell line (DMF=1.2; *P*<0.01) (Figure 3C, D).

### Cell cycle changes with single and combination treatment

The greatest difference in the radiosensitising effect of gemcitabine between the cell lines was seen at 0.3 *μ*M rather than at 0.01 *μ*M, and the former higher dose level was therefore used to study cell cycle effects.

In both cell lines after irradiation, no G1/S block occurred and cells accumulated in G2/M (Table 1A, B

**Table 1:** Cell cycle changes after treatment with gemcitabine (0.3 *μ*M) (4-h incubation) and/or irradiation (2 Gy) in MGH-U1 (A) and S40b (B) cell lines

		**G1**		**S**		**G2/M**	
**Treatment**	**Time (h)**	**% Change**	**± s.e.**	**% Change**	**± s.e.**	**% Change**	**± s.e.**
*(A) MGH-U1*							
Irradiation alone							
	0	−2	1.5	2	1.3	0	0.5
	4	−14	2.8	3	1.1	11	1.9
	8	−11	9.5	−13	5.0	24	4.5
	12	8	1.9	−15	0.8	7	1.7
	16	1	2.3	0	2.8	−2	0.6
	24	1	3.1	−6	2.6	5	1.2
	36	−2	0.8	−2	2.5	3	2.9
	48	0	2.3	−2	2.2	2	3.4
Gemcitabine alone							
	0	−2	1.5	2	1.3	0	0.5
	4	19	1.4	−5	1.3	−14	0.1
	8	9	11.2	−3	3.4	−8	7.9
	12	24	1.4	−9	2.1	−15	0.7
	16	24	0.4	−10	1.5	−14	1.6
	24	21	1.4	−3	1.2	−18	0.3
	36	18	0.6	−3	1.2	−15	0.6
	48	20	2.4	−6	2.4	−14	1.9
Gemcitabine/irradiation							
	0	22	3.4	−6	3.3	−16	0.4
	4	22	3.5	−8	2.7	−14	1.4
	8	18	11.7	−9	3.0	−9	8.9
	12	21	1.3	−4	1.5	−18	0.3
	16	16	1.8	−1	2.2	−15	0.4
	24	17	3.7	−3	4.0	−14	0.3
	36	15	1.0	0	1.3	−14	1.9
	48	16	1.7	0	3.6	−15	2.5
							
*(B) S40b*							
Irradiation alone							
	0	−1	5.7	3	3.1	−1	4.6
	4	−9	2.6	7	1.8	2	1.9
	8	−27	5.4	1	3.3	26	2.2
	12	−13	3.8	−6	1.3	19	3.3
	16	−1	5.5	−7	5.9	9	2.8
	24	1	4.1	1	1.9	−3	2.7
	36	1	4.4	−4	3.2	3	1.3
	48	−4	3.7	1	3.2	3	0.5
Gemcitabine alone							
	0	−1	5.7	3	3.1	−1	4.6
	4	18	7.5	2	1.0	−20	7.9
	8	10	2.1	3	1.9	−13	0.2
	12	12	3.5	−1	2.1	−11	2.3
	16	19	1.5	−7	2.2	−12	1.5
	24	−21	10.9	34	9.4	−12	2.3
	36	−26	4.0	36	6.8	−10	3.1
	48	−26	6.2	31	1.6	−4	7.8
Gemcitabine/irradiation							
	0	14	3.2	−3	1.4	−11	2.4
	4	16	2.9	−5	1.5	−12	1.5
	8	18	1.9	−5	1.6	−14	0.2
	12	−14	7.9	27	9.1	−13	2.0
	16	−39	8.6	46	5.3	−7	3.7
	24	−13	2.7	19	5.9	−6	3.2
	36	−15	4.8	21	5.8	−5	2.6
	48	−13	5.9	16	8.9	−2	3.4

In combination experiments, gemcitabine was given 12 h prior to irradiation. The figures represent the percentage change in the proportion of cells in each part of the cell cycle in comparison to untreated controls. Negative figures represent a reduction in the proportion of cells. Data are obtained from three independent experiments.

). A G2/M block was noted: this peaked at 8 h in both S40b and MGH-U1 and persisted for 12 and 8 h for S40b and MGH-U1, respectively.

When treated with gemcitabine alone (0.3 *μ*M; 4-h incubation), both cell lines exhibited a G1/S block (Table 1A, B). In S40b, the peak occurred at 4 h and the duration was approximately 12 h after which there was an accumulation of cells in the S phase. In contrast, MGH-U1 showed a peak at 12 h and the G1/S block was still present at 48 h.

With both irradiation and gemcitabine (0.3 *μ*M; 4-h incubation, 12 h preirradiation), the G1/S block was observed again (Table 1A, B). The data suggest that the changes observed are similar to those from gemcitabine alone. While studying the duration of the blocks, it should be noted that gemcitabine was given 12 h prior to the first data point in the combination experiments thus showing that the G1/S block in S40b lasted 20 h, while in MGH-U1 it was still present 60 h post gemcitabine. It was observed that in S40b, there was a subsequent accumulation of cells in the S phase; this did not occur in more radiosensitised MGH-U1 cells.

### p53 functional analysis

p53 expression was 90 (s.e.±4) and 182 (s.e.±24) fluorescence units for MGH-U1 and S40b, respectively (*P*=0.12). Cell cycle analysis after 2 Gy in both cell lines showed no G1/S block, suggesting that the p53 was mutant in both cell lines (Table 1A, B).

## DISCUSSION

The ability to improve organ-preserving treatments for bladder cancer hinges on adequate preclinical evaluation. This is in order to predict possible clinical responses, suggest scheduling and also to report the possible mechanisms of action that may aid future treatment selection programmes. The aim of the current study was to identify the radiosensitising effect of gemcitabine on TCC cell lines using the conventional colony-forming assay. We chose two related cell lines with differing radiosensitivities and studied the effects in relation to p53 status and cell cycle perturbations in order that if any difference was elucidated, then it could lead to possible mechanisms of action, which to date are not fully explained for this agent.

The present study shows that low doses of gemcitabine radiosensitise only the S40b cell line while higher doses radiosensitise S40b and MGH-U1. The results suggest that gemcitabine's mechanism of action may depend on the concentration of the drug itself and the presence or absence of certain cellular stress response signals. We previously studied the radiation stress response elements of these two cell lines (Kassem *et al*, 2002) using the Atlas human stress cDNA array™. Of 234 genes blotted on the array, 14 (6%) showed a difference in S40b relative to MGH-U1. In all, 12 genes were down regulated in S40b (HSP90, HSP27, HSP47, heat shock transcription factor 1, NNMT, vimentin, calreticulin precursor, transformation sensitive protein, CDKN1A, GADD153, FLAP endonuclease 1 and NADH-cytochrome *b*5 reductase) and two genes were upregulated (MAPKK2 and MAPKK5). Whether any of these could be responsible for the differences in response observed in this study is debatable and certainly requires further evaluation.

Previous studies on bladder tumour cell lines have produced conflicting results regarding the ability of gemcitabine to radiosensitise (Fechner *et al*, 2003; Pauwels *et al*, 2003). Fechner *et al* studied RT112 (p53 wild type), RT4 (p53 wild type), T24 (p53 mutant) and SUP (p53 mutant) cell lines and found no radiosensitising effect despite gemcitabine (10–500 nM) incubation times of 24 h. In that study, the MTT assay was used, which can provide results for radiation growth delay that are often comparable to those obtained from the colony-forming assay, but it does give more variable and potentially inaccurate results if the assay is not optimised. Furthermore, the assay is affected by the radiation dose level, the concentration of MTT and the duration of the MTT incubation (Price and McMillan, 1990; Slavotinek *et al*, 1994; Banasiak *et al*, 1999). Also, it is known that for survival to be accurately assessed after radiation exposure, the cell population should undergo at least five to six doublings (Steel, 1997). In Fechner *et al*'s study, cells were incubated for only 3 days after treatment during which time they would likely not have had time to exhibit the full effect of radiation. This may have resulted in an underestimate of cell kill and any radiosensitising effect. Pauwels *et al* (2003) studied various cell lines including the ECV304 bladder tumour cell line. In contrast to Fechner *et al*'s study, they showed that a 24-h incubation with gemcitabine (1–6 nM) radiosensitised the bladder tumour cell line with enhancement factors of up to 3.05. They utilised the SRB assay, which again has been shown to provide results comparable to those obtained from the colony-forming assay (Banasiak *et al*, 1999). Unlike Fechner, they optimised the assay for each cell line used and also allowed treated cells to grow for 8 days, thereby allowing the full effect of radiation treatment to be expressed.

The data reported indicate that two related bladder cancer cell lines with different radiosensitivities and similar p53 status are radiosensitised to different extents, in relation to each other, by pretreatment with gemcitabine. This suggests that p53 status is not a dominant factor in the radiosensitising effect of gemcitabine and this is supported by previous reports by Robinson and Shewach (2001) using MCF-7 and MCF-7/Adr breast cancer cell lines and Chen *et al* (2000) using RKO-P and RKO-E6 colon cancer cell lines.

The p53 protein is a tumour suppressor gene involved in the regulation of the cell cycle largely around the G1/S checkpoint. Although p53 status has been shown to be unrelated to gemcitabine radiosensitisation, the data do suggest that other factors around the G1/S checkpoint may be important in the radiosensitising effect of dFdC. Previous studies have highlighted the importance of S-phase accumulation as an important factor in the radiosensitising effect of gemcitabine (Ostruszka and Shewach, 2000). However, our results show that the more radiosensitised cell line (MGH-U1) fails to produce S-phase accumulation. Chen *et al* (2000) showed that p53 wild-type RKO cells were radiosensitised and showed S-phase accumulation, whereas p53 null RKO cells, although showing radiosensitisation, did not accumulate in S phase. The current data show that the p53 mutant MGH-U1 cells did not show S-phase accumulation, but its related p53 mutant S40b did, despite both being radiosensitised by gemcitabine. These contradicting data would suggest that S-phase accumulation is unlikely to be a major mechanism of radiosensitisation by gemcitabine.

The G1 arrest exhibited at 0.3 *μ*M of gemcitabine in combination with irradiation in this study was persistant in MGH-U1 for the duration investigated but in S40b lasted approximately 20 h. The corresponding radiosensitising effect was greater in MGH-U1 than in S40b. The stasis of cells in G1 may result in reduced DNA replication and possibly repair, which may allow the effects of radiation to be greater, as in MGH-U1. However, when cells begin to recycle, it allows them to reinitiate DNA synthesis and repair thereby giving stressed cells a greater ability to recover. This might explain the lower degree of radiosensitisation observed in S40b. The cell cycle effects of gemcitabine were studied extensively by Cappella *et al* (2001). They concluded that the effect of gemcitabine on the cell cycle occurs in two phases. Firstly, a cytostatic phase that results in G1 arrest and secondly, a phase of recycling and DNA synthesis in incompletely recovered cells. This second phase holds the fate of cells and is balanced by DNA synthesis and apoptosis. Our results would support these findings. However, Cappella and co-workers also showed that a second treatment by gemcitabine or cisplatinum potentiated cell kill, if it was given when cells were in the second phase. The model used in our study would not predict this finding. Although we only studied cell cycle changes at the IC_50_ dose, we can postulate that it is likely that, regarding radiosensitisation with gemcitabine, the presence and duration of the cytostatic phase (G1 arrest) and the ability of the cells to replicate or repair DNA is of importance. Furthermore, workers (Merlin *et al*, 1998; Cappella *et al*, 2001) have suggested that p53 is involved only in the cytotoxic rather than the cytostatic phase, which would support our findings that p53 is unimportant in the radiosensitising effect of gemcitabine.

An attractive property of the radiosensitising effect of gemcitabine is that it occurs at noncytotoxic doses. This study shows that at noncytotoxic doses of gemcitabine, only one of the bladder tumour cell lines (S40b) is significantly radiosensitised, whereas both cell lines were radiosensitised when cytotoxic doses were administered. Phase I and II clinical studies of chemoradiation with gemcitabine have reported maximum recommended doses of 300–400 mg m^−2^ in a once-weekly schedule (Pattaranutaporn *et al*, 2001; Wolff *et al*, 2001). These doses are low in comparison to the standard dose of 1000–1200 mg/m^2^ (Stadler *et al*, 1997; von der Maase *et al*, 2000) and are likely to be relatively noncytotoxic. Our results suggest that at such low doses radiosensitisation may be slight and much higher doses may be required, although this would be at the expense of normal tissue toxicity. A method of bypassing this problem and achieving possibly better results would be to adopt a trial design in which gemcitabine is given in its standard dose of 1000 mg m^−2^ and for the radiation dose to be initially reduced prior to escalation. Chen *et al* (2000) also postulated this and they are currently undertaking such trials for pancreatic cancer.

In conclusion, the fluorinated pyrimidine gemcitabine has the potential to improve organ-preserving cancer treatments. It has differential radiosensitising properties in bladder cancer cell lines *in vitro* and while G1 arrest is likely to be important, its effects are certainly unrelated to p53 status. It is likely that there are several factors involved in radiosensitising properties of gemcitabine. A greater understanding of its mechanism of action may aid patient selection. Further work should be directed at investigating the differences in cellular response, between related tumour cells, to this combination treatment.
